# Synergistic Antioxidant Effects of Araloside A and L-Ascorbic Acid on H_2_O_2_-Induced HEK293 Cells: Regulation of Cellular Antioxidant Status

**DOI:** 10.1155/2021/9996040

**Published:** 2021-07-09

**Authors:** Yaqin Tian, Xiuling Zhang, Meiling Du, Fengfeng Li, Manyu Xiao, Wentao Zhang

**Affiliations:** College of Food Science, Northeast Agricultural University, Harbin, Heilongjiang 150030, China

## Abstract

Araloside A is a pentacyclic triterpenoid saponin, and L-ascorbic acid is a globally recognized antioxidant. In this study, coadministered araloside A and L-ascorbic acid were found to have a strong synergistic antioxidant effect, and correlations between cellular antioxidant indexes and free radical scavenging ability were found. Individual and combined pretreatment with araloside A and L-ascorbic acid increased both cell viability and antioxidant enzyme activity and inhibited the release of lactate dehydrogenase (LDH); the accumulation of malondialdehyde (MDA), lipid peroxidation (LPO) products, and H_2_O_2_; and the production of intracellular reactive oxygen species (ROS), protein carbonyls, and 8-hydroxy-2-deoxy guanosine (8-OHdG). Free radical scavenging ability was positively correlated with superoxide dismutase (SOD) and catalase (CAT) activity, the glutathione (GSH)/oxidized glutathione (GSSG) ratio, and total antioxidant capacity (T-AOC). Our study is the first investigation of araloside A and L-ascorbic acid coadministration for the treatment of diseases caused by oxidative stress. The synergistic antioxidant effects of araloside A and L-ascorbic acid support their potential as functional food ingredients for the elimination of oxidative stress-induced adverse reactions.

## 1. Introduction

Selye first proposed the concept of oxidative stress in 1936 [1]. Oxidative stress is essentially caused by an imbalance between excessive free radicals, reactive oxygen species (ROS), reactive nitrogen species (RNS), and weakened antioxidant defence systems [2, 3]. Low-level oxidative stress is a normal process for maintaining life, but excessive oxidative stress causes damage to biomolecules, which leads to the development of certain diseases [4]. H_2_O_2_ not only transmits redox signals under physiological conditions (1-10 nM) but also causes oxidative damage to cells when its concentration exceeds 100 nM [5]. Exogenous H_2_O_2_ can penetrate the cell membrane to induce the production and accumulation of ROS; react with metal ions in the cell; and cause DNA damage, mitochondrial dysfunction, and even H_2_O_2_-mediated apoptosis. The H_2_O_2_-induced cellular oxidative stress model is commonly used to study the functions of various biologically active materials [6, 7]. Therefore, the antioxidant capacities of araloside A, L-ascorbic acid, and their combination were investigated in a human embryonic kidney cell line (HEK293 cells) using H_2_O_2_-induced oxidative stress, which has been utilized in a large number of previous studies. Traditional chemical methods can rapidly detect the antioxidant capacity of active substances but do not reflect the biological behaviour of active substances and downstream molecular mechanisms in living cells. Therefore, the present study is the first investigation of the in vitro and intracellular antioxidant activities of araloside A and L-ascorbic acid and the correlation between these compounds.

HEK293 cells are immortalized cells characterized by normal kidney function with apical zona occludens and relatively inconspicuous brush borders. The metabolic conditions and physiological characteristics of HEK293 cells are closer to those of normal cells than those of cancer cells. These characteristics are more reflective of the true state of oxidative stress, and many researchers have also used HEK293 cells as a cell model of oxidative stress [8, 9].

**Figure 1:**
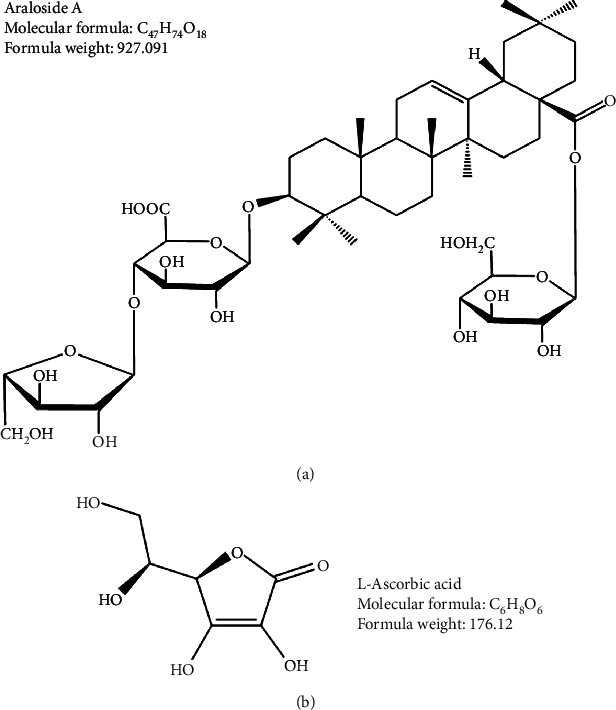
Chemical structure of araloside A (a) and L-ascorbic acid (b).

Triterpenoid saponins, an important class of bioactive products, are primarily divided into tetracyclic triterpenoid saponins and pentacyclic triterpenoid saponins, among which pentacyclic triterpenes are common. Oleanane-type pentacyclic triterpenoid saponins are broadly distributed and have been widely studied [10, 11]. Araloside A (Figure 1), an oleanolic acid-type pentacyclic triterpenoid saponin, is extensively distributed in many Aralia plants, such as *Aralia elata* [12], *Aralia taibaiensis* [13], *Aralia armata* [14], *Aralia chinensis L.* [15], and *Aralia spinifolia* [16]. The mechanism of araloside A absorption in the human body involves multiple transport pathways and mechanisms, such as passive diffusion, paracellular pathways, and mechanisms involving the participation of efflux transporters [17]. Araloside A at doses of 50 and 100 mg/kg was shown to significantly alleviate HCl·ethanol-induced gastric injury and aspirin-induced gastric ulcer [12]. Ding et al. [18] reported that araloside A effectively alleviated inflammation in rheumatoid arthritis fibroblast-like synoviocytes via inhibition of the nuclear factor kappa B pathway. L-Ascorbic acid is a water-soluble vitamin and the essential micronutrient in the human body. At normal physiological concentrations, L-ascorbic acid plays an important role in protecting cells from oxidative damage at normal physiological concentrations, and at higher pharmacological concentrations, it prevents cancer [19]. Araloside A and L-ascorbic acid are widespread in common foods, including fruits, vegetables, herbs, and other plant-based foods. These active ingredients in the daily diet exert antioxidant interactions in the body when ingested simultaneously. These antioxidant interactions may be synergistic, antagonistic, or additive depending on the ratio at which these ingredients are mixed [20]. The antioxidant effects of different active ingredients have been a research focus for many scientists in recent years. However, few scholars have studied the synergistic effects of araloside A and L-ascorbic acid on antioxidant activity.

The objective of this study was to investigate the independent and combined effects of araloside A and L-ascorbic acid on oxidative stress injury in HEK293 cells by monitoring cell viability, antioxidant enzyme capacity, ROS production, lactate dehydrogenase (LDH) release, H_2_O_2_ levels, the extent of lipid peroxidation (LPO), malondialdehyde (MDA) levels, protein carbonyl levels, and 8-hydroxy-2-deoxy guanosine (8-OHdG) levels. In addition, the chemical antioxidant activities of araloside A and L-ascorbic acid were evaluated, and correlations between parameters related to cell antioxidant status and chemical antioxidant activity were analysed.

## 2. Materials and Methods

### 2.1. Materials and Reagents

Araloside A and L-ascorbic acid at a purity ≥98% were purchased from Shanghai Yuanye Biotechnology Co. Ltd. (Shanghai, China). H_2_O_2_ solution (30%) was purchased from Tianli Chemical Reagent Co. Ltd. (Tianjin, China). Minimal essential medium (MEM), penicillin-streptomycin (PSS) solution, foetal bovine serum (FBS), trypsin, RIPA lysis buffer, fluorescence probe (DCFH-DA), phosphate-buffered saline (PBS), 1,1-diphenyl-2-picrylhydrazyl (DPPH), and 2,2′-azino-bis(3-ethylbenzothiazoline-6-sulphonic acid) diammonium salt (ABTS) were obtained from Sigma Chemicals Co. (St. Louis, MO, USA). GoTaq Green Master Mix and CellTiter 96® AQueous One Solution Cell Proliferation Assay (MTS) were purchased from Promega Biotechnology Co. (Madison, WI, USA). Kits used to assay LDH, MDA, LPO, H_2_O_2_, protein carbonyl levels; superoxide dismutase (SOD), glutathione peroxidase (GSH-Px), and catalase (CAT) activities; total antioxidant capacity (T-AOC); and the glutathione/oxidized glutathione (GSH/GSSG) ratio were purchased from Nanjing Jiancheng Bioengineering Co. Ltd. (Nanjing, Jiangsu, China). ELISA kit was purchased from AmyJet Scientific Co. Ltd. (Wuhan, Hubei, China). Xanthine oxidase, *β*-nicotinamide adenine dinucleotide, and lactate dehydrogenase were obtained from Shanghai Macklin Biochemical Co. Ltd. (Shanghai, China).

### 2.2. Cell Culture and Treatment

HEK293 cells were purchased from the Cell Bank of Type Culture Collection of Chinese Academy of Sciences (Shanghai, China) and cultured in MEM supplemented with a 1% PSS and 10% FBS at 37°C in an incubator with 5% CO_2_. The HEK293 cells (5 − 6 × 10^3^ cells/mL) were seeded into flat-bottomed plates and cultured for 24 h in an incubator with CO_2_. When the cells reached approximately 70-80% confluence, the medium was removed, and the cells were pretreated with araloside A (1, 10, or 20 *μ*M), L-ascorbic acid (1, 10, or 20 *μ*M), or both (combined ratio of 1 : 1) for 24 h. Then, the medium was gently removed, and the cells were exposed to H_2_O_2_ for 4 h.

### 2.3. Establishment of the H_2_O_2_-Induced Oxidative Stress Model

HEK293 cells were treated with H_2_O_2_ at different concentrations for 4 h to establish an oxidative stress model. The appropriate amount of H_2_O_2_ was added to medium deprived of serum and antibiotics, and the final concentration of H_2_O_2_ was maintained at 100, 200, 300, 400, 500, 600, 700, or 800 *μ*M. Complete medium without cells served as a blank group, and HEK293 cells without H_2_O_2_ were used as a control group. Cell viability was measured using the MTS assay. MTS (20 *μ*L) was added to the cells, which were then incubated at 37°C for 4 h. The 96-well plate was placed in a multimode microplate reader (Tecan, Infinite M200 Pro) to measure the absorbance at 490 nm. Six parallel experiments with cells in each treatment group were performed, and the results are expressed as average values. All the assays were repeated three times. The concentration of H_2_O_2_ (producing a cell viability of approximately 50%) used to establish the oxidative stress model was selected for subsequent experiments. Cell viability was calculated using the following equation:

(1) $$\begin{array}{c} \text{Cell viability}\left(\%\right)=\frac{{\text{OD}}_{\text{injury group}}-{\text{OD}}_{\text{blank group}}}{{\text{OD}}_{\text{control group}}-{\text{OD}}_{\text{blank group}}}. \end{array}$$

### 2.4. Assessment of the Effects of Araloside A and L-Ascorbic Acid on Cytotoxicity/Proliferation

Normal cells without treatment were regarded as the control group. The cells in the experimental groups were treated with araloside A (1~100 *μ*M) and L-ascorbic acid (1~100 *μ*M) for 24 h at 37°C with 5% CO_2_. Cell viability was measured using the MTS assay. Six parallel experiments with cells in each treatment group were performed, and all assays were repeated at least three times.

### 2.5. Effects of Araloside A and L-Ascorbic Acid at Different Ratios on Cell Viability

Normal cells without treatment were used as the control group, and H_2_O_2_-treated cells served as the injury group. Cells treated with araloside A and L-ascorbic acid at different ratios (1 : 1, 1 : 2, 2 : 1, 1 : 3, or 3 : 1) constituted the synergy groups. HEK293 cells were treated with araloside A and L-ascorbic acid at different ratios for 24 h. The medium was then gently removed, and the cells were exposed to H_2_O_2_ (400 *μ*M) for an additional 4 h. Cell viability was measured using the MTS assay. Six parallel experiments with cells in each treatment group were performed, and all assays were repeated at least three times.

### 2.6. Protective Effects of Araloside A, L-Ascorbic Acid, and the Combination on Cell Viability

Normal cells without treatment were used as the control group, and H_2_O_2_-treated cells constituted the injury group. Cells in the experimental groups were treated with araloside A (1, 10, or 20 *μ*M), L-ascorbic acid (1, 10, or 20 *μ*M), or the combination (ratio of 1 : 1) for 24 h at 37°C in the presence of 5% CO_2_. The cells were treated with H_2_O_2_ (400 *μ*M) for an additional 4 h, and cell viability was measured using the MTS assay. Six parallel experiments with cells in each treatment group were performed, and all assays were repeated at least three times.

### 2.7. Measurement of Intracellular ROS Production

A DCFH-DA fluorescent probe assay was used to determine intracellular ROS production. Incomplete medium containing DCFH-DA (10 *μ*M) was added to the cells, which were then incubated at 37°C for 20 min. The fluorescent probe (DCFH-DA) was removed, and the cells were washed three times with PBS. The green fluorescence from DCF in the HEK293 cells was determined by fluorescence microscopy (BX43 upright microscope, Olympus, Long Island, New York, USA). Fluorescence intensity was immediately detected using a multimode microplate reader with excitation wavelength of 485 nm and an emission wavelength of 525 nm. All assays were repeated at least three times.

### 2.8. Measurement of Relevant Indicators of Cell Antioxidant Status

Cells digested with trypsin were washed twice with PBS. The cell pellet remaining after centrifugation was lysed in ice-cold RIPA lysis buffer containing 1 mM PMSF for 40 min. The relevant indicators were determined according to the instructions provided with the corresponding assay kits, and the principles of each biochemical methods were as follows. SOD activity (superoxide dismutase assay kit, A001-3) was determined via colorimetric analysis of 2-(4-iodophenyl)-3-(4-nitrophenyl)-5-(2,4-disulfophenyl)-2H-tetrazolium formazan at 450 nm. GSH-Px activity (glutathione peroxidase assay kit, A005-1) was determined by colorimetric analysis via measurement of the consumption of GSH at 412 nm. CAT activity (catalase assay kit, A007-1) was determined by measuring the decomposition of H_2_O_2_ via absorbance at 405 nm. T-AOC was estimated by measuring the absorbance of ABTS^+^ at 415 nm. The cyclic reaction of 5,5′-dithiobis-(2-nitrobenzoic acid) was used to determine the total glutathione and GSSG levels (total glutathione/oxidized glutathione assay kit, A061-1). The GSH content was equal to the total glutathione content minus 2 times the GSSG content. The MDA level (cell malondialdehyde assay kit, A003-4) was analysed colorimetrically by measuring condensation of thiobarbituric acid at 523 nm. The H_2_O_2_ level (hydrogen peroxide assay kit, A064-1) was evaluated by measuring the amount of the complex formed by the reaction between H_2_O_2_ and molybdic acid at 405 nm. The LPO content (lipid peroxidation assay kit, A106-1) was analysed by measuring the absorbance following the reaction between LPO and the developer at 586 nm. The protein carbonyl content (protein carbonyl assay kit, A087-1) was determined by measuring the absorbance of 2,4-dinitrophenylhydrazone at 370 nm. The medium was collected, and LDH release (lactate dehydrogenase assay kit, A020-2) was determined by measuring the change in colour of pyruvate-dinitrophenylhydrazone at 450 nm. The amount of 8-OHdG was determined using a standard ELISA kit following the manufacturer's instructions. All assays were repeated at least three times.

### 2.9. Determination of In Vitro Antioxidant Activity

The DPPH assay was performed according to the method described by Fattouch et al. with slight modifications [21]. Samples (2 mL) and 2 mL of a DPPH^·^ solution were reacted at room temperature for 30 min in the dark, and the absorbance at a wavelength of 517 nm was measured and recorded as *A*_*i*_. The sample was replaced with 70% methanol, and the absorbance was measured and recorded as *A*_*c*_. The DPPH^·^ solution was replaced with 70% methanol, and the absorbance was measured and recorded as *A*_*t*_. The DPPH^·^ scavenging ratio was calculated as follows:

(2) $$\begin{array}{c} {\text{DPPH}}^{.}\text{ scavenging ratio}\left(\%\right)=\left(1-\frac{A_{i}-A_{t}}{A_{c}}\right)\times 100\%. \end{array}$$

The ABTS assay was performed as described by Re et al. [22]. Three millilitres of ABTS^+^ solution and 100 *μ*L of sample were mixed at 30°C for 6 min in the dark, and the absorbance at 743 nm was measured and recorded as *A*_*i*_. The sample was replaced with 70% methanol, and the absorbance was measured and recorded as *A*_*c*_. The ABTS^+^ working solution was replaced with 70% methanol, and the absorbance was measured and recorded as *A*_*t*_. The calculations were performed as described above.

Hydroxyl radical scavenging capacity was determined according to Denev et al. with some modifications [23]. The sample (1 mL) was added to 1 mL of an FeSO_4_ solution (10 mmol/L), 1 mL of a salicylic acid solution (10 mmol/L), and 1 mL of H_2_O_2_ (6 mmol/L). The mixture was cooled rapidly, and the absorbance at 510 nm was measured and recorded as *A*_*i*_. The sample was replaced with 70% methanol, and the absorbance was measured and recorded as *A*_*c*_. The FeSO_4_ solution was replaced with 70% methanol, and the absorbance was measured and recorded as *A*_*t*_. The calculations were performed as described above.

Superoxide anion radical scavenging capacity was determined according to Chan and Bielski with some modifications [24]. The sample (200 mL) was added to 1 mL of sodium phosphate buffer (pH 7.0), 500 *μ*L of *β*-nicotinamide adenine dinucleotide, and 100 *μ*L of lactate dehydrogenase and allowed to react at 25°C for 1 min. Xanthine oxidase (10 mL) was then added, and the absorbance at 340 nm was measured and recorded as *A*_*i*_. The absorbance of an equal volume of distilled water (instead of the sample) was measured and recorded as *A*_*c*_, and the absorbance of an equal volume of distilled water instead of the pyrogallol solution was measured and recorded as *A*_*t*_. The calculations were performed as described above. All assays were repeated at least three times.

### 2.10. Assessing the Synergistic Effect of Araloside A and L-Ascorbic Acid in Combination by the Isobologram Method

The antioxidant interaction of araloside A and L-ascorbic acid was evaluated by the isobologram method according to Jiang et al. [25]. The synergistic rate (SR) was calculated by the equation: SR = EV/TV, where EV is the experimental value of the combination group and TV is the theoretical value of the combination group. TV was calculated as follows: TV = *a*∗*R*_*A*_ + *b*∗*R*_*B*_, where *a* and *b* are the combined ratios of substance *A* and substance *B*, and *R*_*A*_ and *R*_*B*_ are the experimental results obtained when substance *A* and substance *B* are used separately. SR > 1 indicated synergistic effects, SR < 1 indicated the antagonistic effects, and SR = 1 indicated additive effects. In this study, the SRs based on LDH release, ROS generation, GSSG content, MDA content, H_2_O_2_ level, LPO content, protein carbonyl level, and 8-OHdG level were less than 1, indicating synergistic effects.

### 2.11. Statistical Analysis

All assays were repeated at least three times, and the data are expressed as the mean ± standard deviation. The normality of the distribution was checked using the KURT and SKEW functions in Microsoft Office Excel 2019. Homogeneity of variance was tested with the homogeneity of variance test using SPSS 20.0 (IBM Corp., Armonk, NY, USA). A correction for multiple comparisons was calculated. Correlations between data were assessed with bivariate and Pearson correlation coefficients using SPSS 20.0. The data were subjected to one-way analysis of variance (ANOVA) in SPSS 20.0 to analyse the statistical significance. Significance was established at a level of *p* < 0.05.

## 3. Results

### 3.1. Cytotoxic Effects of H_2_O_2_ in HEK293 Cells

**Figure 2:**
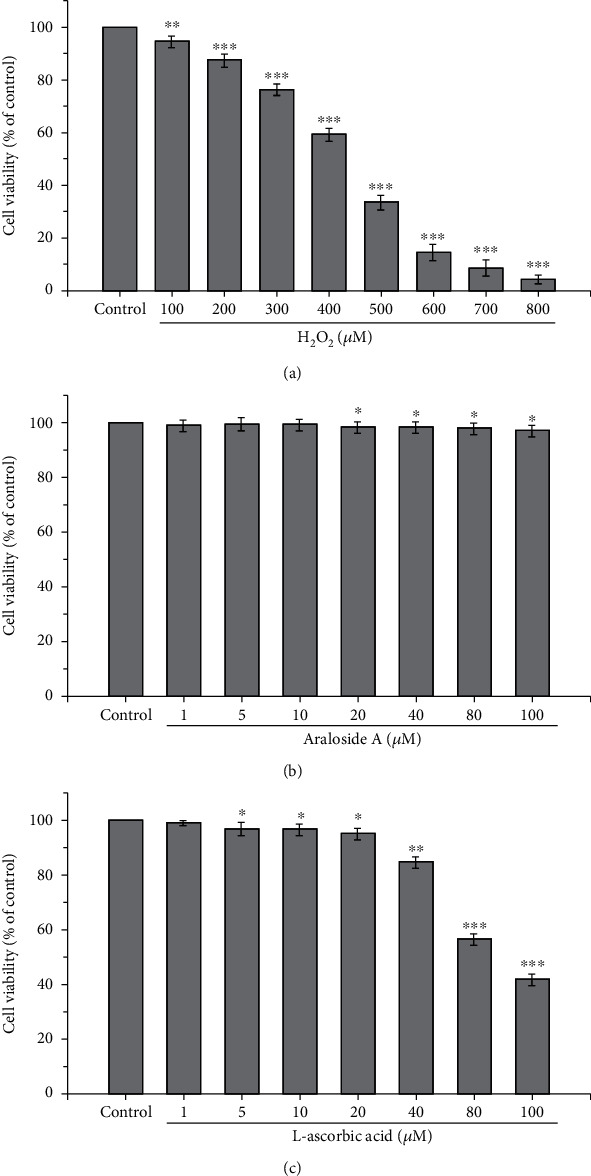
Cytotoxicity effects of H_2_O_2_ (a), araloside A (b), and L-ascorbic acid (c) on the viability of HEK239 cells. ^∗^*p* < 0.05, ^∗∗^*p* < 0.01, and ^∗∗∗^*p* < 0.001 compared with the control group.

HEK293 cells were treated with 100-800 *μ*M H_2_O_2_ for 4 h, and cell viability was measured using the MTS method. As shown in Figure 2(a), cell viability underwent a concentration-dependent decrease with increasing H_2_O_2_ concentration. The cell viability obtained with the minimum treatment concentration (100 *μ*M) and the maximum treatment concentration (800 *μ*M) was 94.45% and 3.91%, respectively. Treatment with 400 *μ*M H_2_O_2_ reduced cell viability to approximately 41% of that of the control group. Therefore, 400 *μ*M H_2_O_2_ was selected as the optimal concentration to establish the oxidative stress model in HEK293 cells in the subsequent experiments.

### 3.2. Cytotoxic Effects of Araloside A and L-Ascorbic Acid

In this study, a cell viability between 95% and 105% indicated no cytotoxicity or proliferation of HEK293 cells. Araloside A did not significantly enhance or inhibit the viability of HEK293 cells when its concentration was below 100 *μ*M (Figure 2(b)). L-Ascorbic acid did not significantly enhance or inhibit the viability of HEK293 cells when its concentration was below 20 *μ*M (Figure 2(c)). Moreover, the plasma concentrations of L-ascorbic acid following food intake do not exceed 100 *μ*M [26]. Considering that the actual plasma concentration of araloside A is relatively low and that physiological araloside A concentrations in previous studies did not exceed 20 *μ*M [18], L-ascorbic acid and araloside A concentrations were maintained under 20 *μ*M for further experiments.

### 3.3. Effects of Araloside A and L-Ascorbic Acid at Various Ratios on Cell Viability

**Figure 3:**
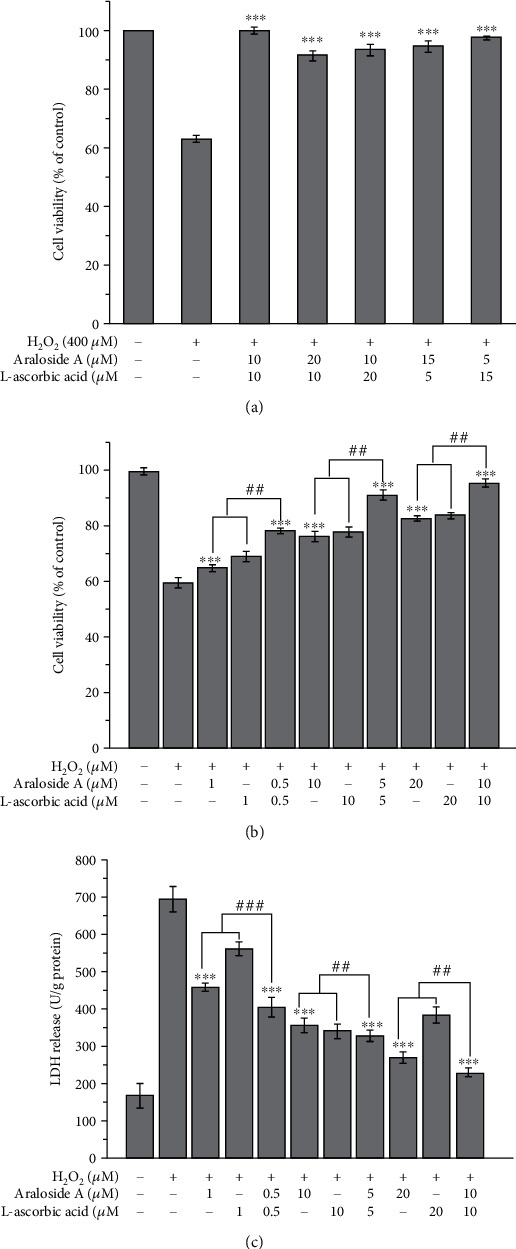
Individual and combined effect of araloside A and L-ascorbic acid on HEK293 cells. (a) The effects of araloside A and L-ascorbic acid with variously combined ratio on H_2_O_2_-induced oxidative damage in HEK293 cells. (b) Protective effect of araloside A and L-ascorbic acid on H_2_O_2_-induced oxidative damage in HEK293 cells. (c) Individual and combined effect of araloside A and L-ascorbic acid on lactate dehydrogenase (LDH) release. Vertical bars indicate the mean value ± SD. ^##^*p* < 0.01 and ^###^*p* < 0.001 versus the araloside A+L-ascorbic acid group. ^∗∗∗^*p* < 0.001 versus the injury group.

The inhibitory effects of araloside A and L-ascorbic acid in combination at various ratios on the induction of oxidative stress-related damage were evaluated using the MTS assay. After the coadministration of araloside A and L-ascorbic acid at a ratio of 1 : 1, the cell viability reached 101.01 ± 1.10%, which was 39.10% higher than that obtained in the injured group and 9.5%, 8.44%, 7.49%, and 3.40% higher than that obtained after their coadministration at ratios of 1 : 2, 1 : 3, 2 : 1, and 3 : 1, respectively (Figure 3(a)). Thus, araloside A and L-ascorbic acid at a ratio of 1 : 1 exerted the best inhibitory effects on H_2_O_2_-induced oxidative stress-related damage.

### 3.4. Individual and Combined Protective Effects of Araloside A and L-Ascorbic Acid against H_2_O_2_-Induced Oxidative Stress

As shown in Figure 3(b), the viability of the injured group was 59.59 ± 1.88%, which was significantly lower than that of the control group. Cell viability after pretreatment with araloside A, L-ascorbic acid, or their combination increased from 71.16 ± 1.05% to 95.44 ± 1.55% compared with that of the injury group. Pretreatment araloside A and/or L-ascorbic acid at higher concentrations increased cell viability. The cell viability obtained with combined araloside A and L-ascorbic acid pretreatment was significantly higher than that obtained upon pretreatment with the individual compounds. According to the isobologram method, the SR at all three concentrations (1, 10, or 20 *μ*M) was greater than 1 (Table 1). The viability of the cells under combination pretreatment with 1/2 araloside A and 1/2 L-ascorbic acid was significantly improved compared with that upon pretreatment with individual araloside A or L-ascorbic acid treatment at the same concentration. These results indicate that the combination of araloside A and L-ascorbic acid significantly enhanced the inhibition of H_2_O_2_-induced oxidative stress damage. Therefore, the combination of araloside A and L-ascorbic acid improved the cytoprotective effect against oxidative damage in HEK293 cells via synergy.

### 3.5. Individual and Combined Effects of Araloside A and L-Ascorbic Acid on LDH Release

The individual and combined protective effects of araloside A and L-ascorbic acid on plasma membrane integrity were evaluated by measuring the release of LDH. As shown in Figure 3(c), LDH release in the control group was significantly lower than that in the injury group. The damage to HEK293 cells induced by H_2_O_2_ resulted in destruction of the plasma membrane and thus a sharp increase in the level of LDH release compared with that of the control group. However, LDH release after pretreatment with araloside A or L-ascorbic acid was significantly reduced compared with that in the injury group, and the combination of araloside A and L-ascorbic acid induced a significantly larger reduction in LDH release compared with that upon pretreatment with either individual compound. The amount of LDH released by the combination group (10 *μ*M araloside A + 10 *μ*M L − ascorbic acid) was 42.13 and 55.04 U/g protein lower than those of the groups treated with 20 *μ*M araloside A and 20 *μ*M L-ascorbic acid alone, respectively. The SR of the group treated with araloside A+L-ascorbic acid (10 *μ*M + 10 *μ*M) was 0.70 ± 0.01, showing the significant synergistic effects of the two compounds (Table 1). These results confirm that araloside A and L-ascorbic acid synergistically stabilize the release of LDH and improve the protective capacity of the cell plasma membrane.

### 3.6. Individual and Combined Effects of Araloside A and L-Ascorbic Acid on H_2_O_2_-Induced ROS Generation

**Figure 4:**
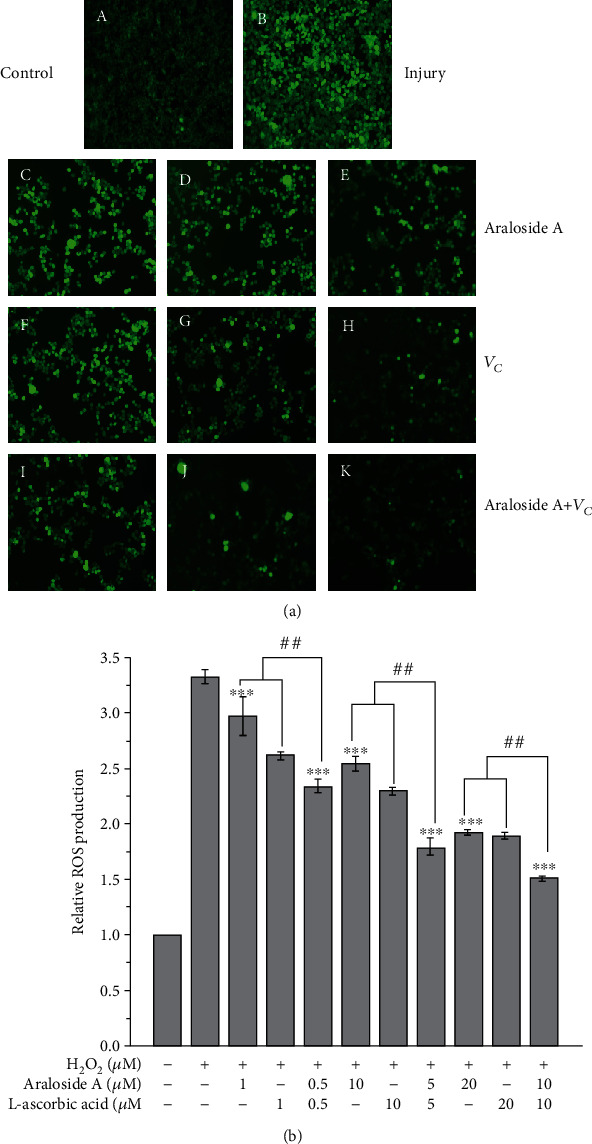
Individual and combined effect of araloside A and L-ascorbic acid (*V*_*C*_) on reactive oxygen species (ROS) generation. (a) Fluorescence images in HEK293 cells were collected by laser scanning confocal microscopy (LSCM) 200x. (A) Control group; (B) injury group; (C) 1 *μ*M araloside A + 400 *μ*M H_2_O_2_; (D) 10 *μ*M araloside A + 400 *μ*M H_2_O_2_; (E) 20 *μ*M araloside A + 400 *μ*M H_2_O_2_; (F) 1 *μ*M L − ascorbic acid + 400 *μ*M H_2_O_2_; (G) 10 *μ*M L − ascorbic acid + 400 *μ*M H_2_O_2_; (H) 20 *μ*M L − ascorbic acid + 400 *μ*M H_2_O_2_; (I) 0.5 *μ*M araloside A + 0.5 *μ*M L − ascorbic acid + 400 *μ*M H_2_O_2_; (J) 5 *μ*M araloside A + 5 *μ*M L − ascorbic acid + 400 *μ*M H_2_O_2_; (K) 10 *μ*M araloside A + 10 *μ*M L − ascorbic acid + 400 *μ*M H_2_O_2_. (b) Production of intracellular ROS was measured using the fluorescence probe 2′,7′-dichlorodihydrofluorescein diacetate (DCFH-DA). Vertical bars indicate the mean values ± SD. ^##^*p* < 0.01 versus the araloside A+L-ascorbic acid group. ^∗∗∗^*p* < 0.001 versus the injury group.

Intracellular DCF fluorescence images showed that the control group exhibited a weak green fluorescence intensity, which indicated that the production and elimination of ROS in HEK293 cells were in equilibrium (Figure 4(a)). The green fluorescence intensity of the injury group was significantly enhanced, implying the rapid generation and accumulation of ROS in the HEK293 cells. However, the green fluorescence intensity after pretreatment with araloside A or L-ascorbic acid was obviously weaker than that of the injury group, and the combination of araloside A and L-ascorbic acid attenuated the green fluorescence intensity to a greater extent than pretreatment with the individual compounds.

The level of ROS generated in the control group was designated 1.0 and used to express relative ROS production in the other groups. The data showed that relative ROS production in the injury group was approximately 2.3-fold higher than that in the control group (Figure 4(b)). Relative ROS production after pretreatment with araloside A, L-ascorbic acid, or their combination was significantly lower than that in the injury group, and ROS production after pretreatments gradually approached the baseline obtained from the control group in a concentration-dependent manner. Notably, the combination of araloside A and L-ascorbic acid significantly decreased relative ROS production compared with that of the individual pretreatment groups. The isobologram method showed that the combination of 5 *μ*M araloside A and 5 *μ*M L-ascorbic acid produced a greater effect than 10 *μ*M araloside A or L-ascorbic acid, which indicated that araloside A and L-ascorbic acid had a synergistic effect (SR = 0.73 ± 0.05, Table 1). These data demonstrated that araloside A and L-ascorbic acid synergistically protected HEK293 cells against H_2_O_2_-induced oxidative stress and that these effects might be due to improvement in the ability to scavenge or inhibit ROS.

### 3.7. Individual and Combined Regulatory Effects of Araloside A and L-Ascorbic Acid on the Antioxidant Status of H_2_O_2_-Exposed HEK293 Cells

**Figure 5:**
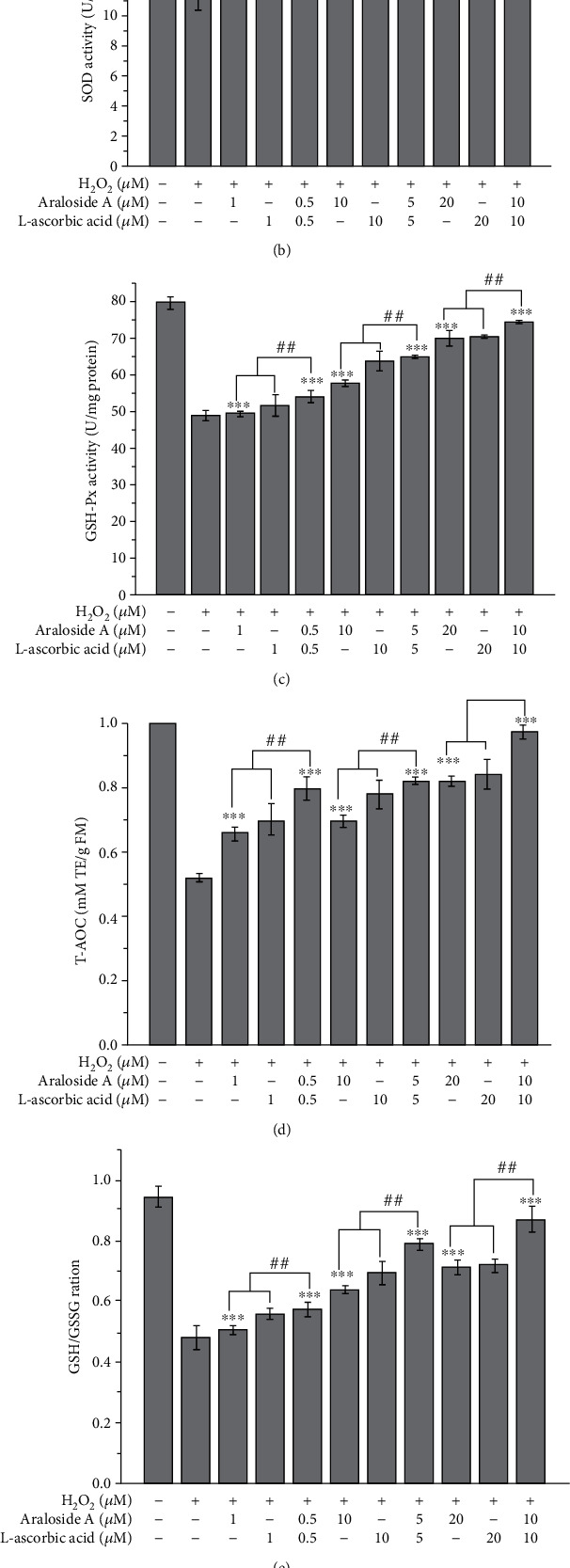
Individual and combined effect of araloside A and L-ascorbic acid on antioxidant status of H_2_O_2_-exposed HEK293 cells. (a) Catalase (CAT) activity. (b) Superoxide dismutase (SOD) activity. (c) Glutathione peroxidase (GSH-Px) activity. (d) Total antioxidant capacity (T-AOC) activity. (e) Glutathione/oxidized glutathione (GSH/GSSG) ratio. (f) Oxidized glutathione (GSSG) levels. Vertical bars indicate the mean values ± SD. ^##^*p* < 0.01 and ^###^*p* < 0.001 versus the araloside A+L-ascorbic acid group. ^∗∗^*p* < 0.01 and ^∗∗∗^*p* < 0.001 versus the injury group.

H_2_O_2_ exposure induces oxidative stress-related damage in HEK293 cells by reducing the activity of antioxidant enzymes and destroying the antioxidant defence system. The individual and combined effects of araloside A and L-ascorbic acid on the activities of antioxidant enzymes (CAT, SOD, and GSH-Px) are shown in Figure 5. CAT, SOD, and GSH-Px activities were significantly decreased in the H_2_O_2_-treated group, but pretreatment with araloside A, L-ascorbic acid, or their combination effectively prevented the H_2_O_2_-induced reduction in antioxidant enzyme activity. However, combined pretreatment with araloside A and L-ascorbic acid inhibited the reduction in antioxidant enzyme activity more effectively than individual araloside A or L-ascorbic acid pretreatment. For example, the SR of SOD was 1.88 ± 0.04 (SR > 1) in the araloside A-L-ascorbic acid group (10 *μ*M), indicating synergistic effects (Table 1). H_2_O_2_ treatment also decreases the T-AOC of HEK293 cells. T-AOC activity decreased by 0.38 mM TE/g FM in the H_2_O_2_-treated group compared with that of the control group. However, the T-AOC after treatment with the combination of araloside A and L-ascorbic acid (10 *μ*M) increased by 0.35 mM TE/g, which was markedly higher than the increases obtained with individual pretreatment. HEK293 cells exposed to H_2_O_2_ exhibited a decreased GSH/GSSG ratio and an increased level of GSSG (Figures 5(e) and 5(f)). Compared with individual pretreatment, combined pretreatment with araloside A and L-ascorbic acid significantly reduced the GSSG content and increased the GSH/GSSG ratio in a concentration-dependent manner. Araloside A and L-ascorbic acid inhibited H_2_O_2_-induced oxidative stress-related damage by increasing antioxidant enzyme activity and maintaining the antioxidant defence system. Table 1 shows that the SRs of antioxidant enzymes, T-AOC, and the GSH/GSSG ratio were all greater than 1. These results further support the presence of a synergistic effect between araloside A and L-ascorbic acid.

### 3.8. Individual and Combined Effects of Araloside A and L-Ascorbic Acid on the MDA Content, LPO Content, and H_2_O_2_ Level

**Figure 6:**
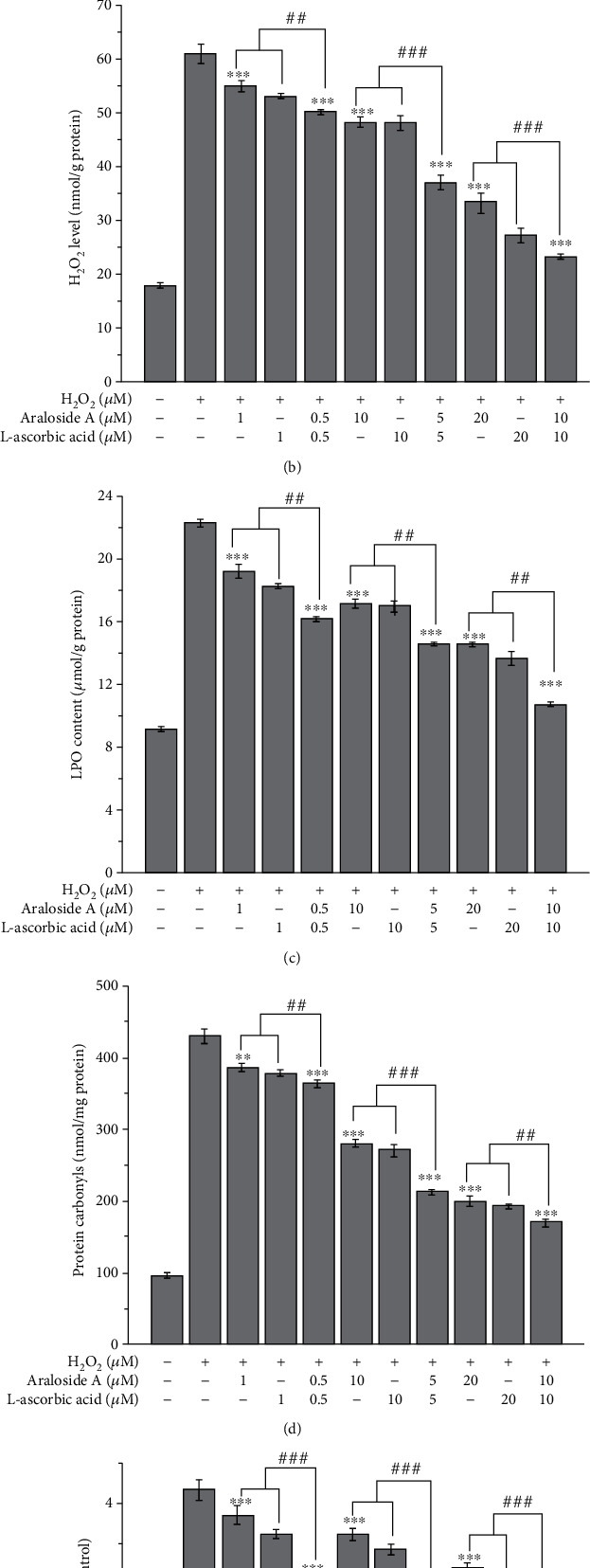
Individual and combined effect of araloside A and L-ascorbic acid on lipid, protein, and DNA damage. (a) Malondialdehyde (MDA) level. (b) H_2_O_2_ level. (c) Lipid peroxidation (LPO) contents. (d) Protein carbonyl levels. (e) 8-Hydroxy-2-deoxy guanosine (8-OHdG) levels. Vertical bars indicate the mean values ± SD. ^##^*p* < 0.01 and ^###^*p* < 0.001 versus the araloside A+L-ascorbic acid group. ^∗∗^*p* < 0.01 and ^∗∗∗^*p* < 0.001 versus the injury group.

To further evaluate the individual and combined effects of araloside A and L-ascorbic acid on H_2_O_2_-induced oxidative stress, the MDA level, LPO content, and H_2_O_2_ level of each treatment group were measured using kits (Figure 6). The MDA level of the control group was 12.26 nmol/mg protein, and H_2_O_2_ exposure significantly increased the MDA level to approximately 4-fold that of the control group. However, the MDA level obtained after pretreatment with araloside A or L-ascorbic acid was significantly lower than that of the injury group, which indicated that pretreatment with araloside A or L-ascorbic acid effectively inhibited the H_2_O_2_-induced production of MDA. In addition, the MDA level in HEK293 cells treated with araloside A combined with L-ascorbic acid was lower than those after individual pretreatment, and this effect was concentration-dependent. Furthermore, the LPO content in the combined pretreatment groups (0.5 *μ*M + 0.5 *μ*M: 16.13 *μ*mol/g protein; 5 *μ*M + 5 *μ*M: 14.55 *μ*mol/g protein; 10 *μ*M + 10 *μ*M: 10.71 *μ*mol/g protein) was also lower than that after individual pretreatment (1, 10, and 20 *μ*M araloside A: 19.17, 17.11, and 14.49 *μ*mol/g protein, respectively; 1, 10, and 20 *μ*M L-ascorbic acid: 18.22, 16.95, and 13.63 *μ*mol/g protein, respectively). Measurement of the H_2_O_2_ levels revealed the same trend after comparison in the same way. This experiment indicated that araloside A and L-ascorbic acid inhibited H_2_O_2_-induced oxidative stress-related damage in HEK293 cells by reducing the degree of lipid oxidation and levels of free radicals in a synergistic manner (SR < 1).

### 3.9. Individual and Combined Protective Effects of Araloside A and L-Ascorbic Acid against Protein and DNA Damage

H_2_O_2_-induced oxidative stress caused varying degrees of oxidative damage to proteins and DNA (Figures 6(d) and 6(e)). Figure 6 shows that the protein carbonyl and 8-OHdG levels in HEK293 cells exposed to H_2_O_2_ were significantly higher than those of the control group. However, the protein carbonyl and 8-OHdG levels after pretreatment with araloside A, L-ascorbic acid, or their combination were significantly reduced compared with those of the injury group. Surprisingly, the reduction in the combined pretreatment group (araloside A+L-ascorbic acid) was more significant than those after individual pretreatment. For example, the SRs for protein carbonyl and 8-OHdG levels in the araloside A-L-ascorbic acid group (10 *μ*M + 10 *μ*M) were 0.77 ± 0.04 and 0.80 ± 0.01 (SR < 1), respectively, indicating an obvious synergistic effect (Table 1). These results demonstrated that araloside A and L-ascorbic acid synergistically protect proteins and DNA from H_2_O_2_-induced damage.

### 3.10. Combined Effects of Araloside A and L-Ascorbic Acid on In Vitro Free Radical Scavenging Capacity

**Figure 7:**
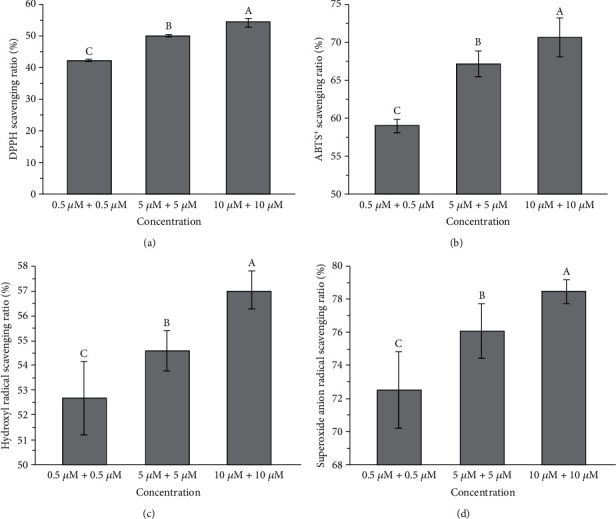
Combined effect of araloside A and L-ascorbic acid on chemical antioxidant capacity. (a) DPPH^·^ free radical scavenging capacity. (b) ABTS^+^ free radical scavenging capacity. (c) Hydroxyl radical scavenging capacity. (d) Superoxide anion radical scavenging capacity. Vertical bars indicate the mean values ± SD. Different letters within each column indicate significant differences at *p* < 0.05.

The combined chemical antioxidant potential of araloside A and L-ascorbic acid was evaluated by measuring the capacity to scavenge DPPH^·^ and ABTS^+^ free radicals, the hydroxyl radical, and the superoxide anion (Figure 7). The results showed that araloside A and L-ascorbic acid induced concentration-dependent increases in antioxidant activity against DPPH^·^ radicals. The DPPH^·^ radical scavenging rate upon combination treatment with 10 *μ*M araloside A and 10 *μ*M L-ascorbic acid was 54.36%, which was significantly higher than that upon treatment of the combination of 0.5 *μ*M araloside A and 0.5 *μ*M L-ascorbic acid. The potential of araloside A and L-ascorbic acid to scavenge ABTS^+^ radicals also increased in a concentration-dependent manner. The effects of combination treatment with araloside A and L-ascorbic acid on the hydroxyl radical and superoxide anion radical scavenging rates followed a trend similar to that shown by the ABTS^+^ radical scavenging rate, and the findings showed that the scavenging rates gradually increased with increasing the pretreatment concentration.

### 3.11. Correlation between Cell Antioxidant Status and Chemical Antioxidant Capacity

**Figure 8:**
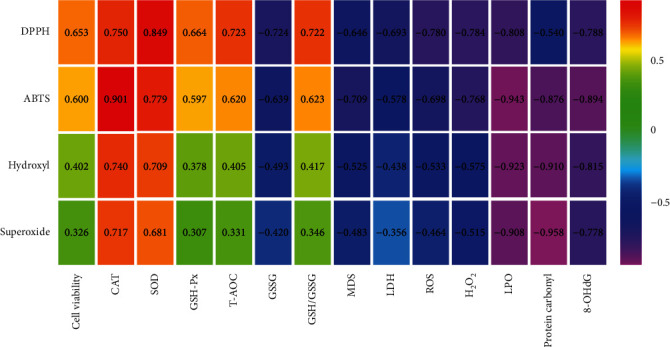
Correlation coefficients of cell antioxidant status and DPPH^·^ radicals scavenging capacity, ABTS^+^ radicals scavenging capacity, hydroxyl radicals scavenging capacity, and superoxide anion radicals scavenging capacity of combination of araloside A and L-ascorbic acid. CAT: the activity of catalase; SOD: the activity of superoxide dismutase; GSH-Px: the activity of glutathione peroxidase; T-AOC: the total antioxidant capacity; GSSG: oxidized glutathione; GSH/GSSG: the glutathione/oxidized glutathione ratio; MDA: the malondialdehyde levels; LDH: the release of lactate dehydrogenase; ROS: the production of reactive oxygen species; H_2_O_2_: the H_2_O_2_ levels; LPO: the lipid peroxidation contents; 8-OHdG: the 8-hydroxy-2-deoxy guanosine levels.

The correlation between chemical antioxidant capacity (the capacity to scavenge DPPH^·^, ABTS^+^, hydroxyl, and superoxide anion radicals) and the antioxidant status of HEK293 cells after combined treatment is shown in Figure 8. CAT activity in H_2_O_2_-injured HEK293 cells after the coadministration of araloside A and L-ascorbic acid was significantly and positively correlated with the capacity to scavenge DPPH^·^, ABTS^+^, hydroxyl, and superoxide anion radicals, with correlation coefficients of 0.750 (*p* = 0.020, <0.05), 0.901 (*p* = 0.001, <0.01), 0.740 (*p* = 0.023, <0.05), and 0.717 (*p* = 0.030, <0.05), respectively. A significant positive correlation between SOD activity and chemical antioxidant capacity was found, with correlation coefficients of 0.849 (*p* = 0.004, <0.01, DPPH), 0.779 (*p* = 0.013, <0.05, ABTS), 0.709 (*p* = 0.032, <0.05, hydroxyl), and 0.681 (*p* = 0.043, <0.05, superoxide anion). No significant correlation between GSH-Px activity and the viability or radical scavenging ability of HEK293 cells was found. In addition, the data showed a positive correlation between T-AOC and DPPH^·^ radical scavenging capacity (*r* = 0.723, *p* = 0.028, <0.05). A significant negative correlation (*r* = −0.724, *p* = 0.027, <0.05) was observed between GSSG content and DPPH^·^ radical scavenging capacity, and a positive correlation (*r* = 0.722, *p* = 0.028, <0.05) was found between the GSSG/GSH ratio and DPPH^·^ radical scavenging capacity. The study of oxidation products revealed significant negative correlations (*r* = −0.709, *p* = 0.032, <0.05, MDA; *r* = −0.943, *p* = 0.001, <0.01, LPO) between the MDA level and LPO content with the ABTS^+^ radical scavenging capacity. Moreover, a negative correlation (*r* = −0.693, *p* = 0.038, <0.05) between LDH release and DPPH^·^ radical scavenging capacity was also found. In addition, free radical levels were negatively correlated with protein and DNA damage (protein carbonyl and 8-OHdG levels). Additionally, the capacity to scavenge DPPH^·^ (*r* = −0.780, *p* = 0.013, <0.05, ROS; *r* = −0.784, *p* = 0.012, <0.05, H_2_O_2_) and ABTS^+^ (*r* = −0.698, *p* = 0.036, <0.05, ROS; *r* = −0.768, *p* = 0.016, <0.05, H_2_O_2_) was negatively correlated with relative ROS production and the H_2_O_2_ level.

## 4. Discussion

Synergistic or antagonistic effects may occur between ingested foods and drugs. Ginsenoside Rg1 and heat shock protein 70 were shown to synergistically inhibit tert-butyl hydroperoxide-induced damage in neural stem cells [27], and synergistic antioxidant effects among vitamin E, epicatechin, and catechin ester acids have been found [28]. In addition, flavanols, pigments (beets, lycopene, and *β*-carotene), and common anthocyanins in foods exhibit synergistic effects on antioxidant activity by synergistically inhibiting free hydrogen peroxide radicals [29]. Relatively few studies have investigated whether araloside A exerts synergistic or antagonistic effects with certain ingredients in food and medicine. In this study, the synergistic antioxidant effects of araloside A and L-ascorbic acid were evaluated using the isobologram method. The results showed that araloside A and L-ascorbic acid significantly inhibited H_2_O_2_-induced oxidative stress in HEK293 cells and exerted synergistic effects to reduce intracellular ROS production, the MDA content, the LPO content, LDH release, H_2_O_2_ levels, the protein carbonyl content, and 8-OHdG levels and increase the activity of related antioxidant enzymes. Moreover, the combination of araloside A and L-ascorbic acid exerted significant effects in the scavenging of DPPH^·^, ABTS^+^, hydroxyl, and superoxide anion radicals. In addition, the inhibitory effects of araloside A and L-ascorbic acid on H_2_O_2_-induced oxidative stress in HEK293 cells were found to be correlated with their ability to scavenge DPPH^·^, ABTS^+^, hydroxyl, and superoxide anion free radicals.

Araloside A can scavenge DPPH^·^, ABTS^+^, hydroxyl, and superoxide anion radicals, proving its potential to act as a prooxidant under certain conditions. Prooxidants at low or moderate concentrations are beneficial for antioxidant defence systems, but at higher concentrations, they act as toxic agents that cause oxidative damage. When the concentration of araloside A exceeded 16 *μ*M or 108 *μ*M, the survival rates of human rheumatoid arthritis fibroblast-like synoviocytes and Caco-2 cells were less than 50% [17, 18]. However, the role of araloside A as a prooxidant should attract attention in cancer therapy. Previous studies have shown that araloside A is an oleanolic acid trioside with glucose, arabinose, and glucuronic acid residues and sugar residues attached to the C-3 and C-28 sites of its mother nucleus (Figure 1(a)) [30]. Yoshikawa et al. suggested that the basic active structure of several oleanosides, such as araloside A, contains a monosaccharide chain at the C-3 position of the mother nucleus [31]. Data from our current study showed that araloside A alleviated H_2_O_2_-induced oxidative stress in HEK293 cells. This result indicated that araloside A might have lost its glucose residues through the hydrolysis of glycosidic bonds and thus achieved partial antioxidant activity. Among a dozen globally recognized vitamins, L-ascorbic acid has the simplest molecular structure (Figure 1(b)). The antioxidant capacity of L-ascorbic acid, an acidic 3-keto-L-furanogluonic acid lactone with an enol-like structure containing six carbon atoms, primarily manifests through its rapid reaction with free radicals, such as O_2_^-·^, HO^·^, and RO^·^. This reaction not only produces semidehydroascorbic acid but also scavenges singlet oxygen and reduces free sulphur radicals [32]. This antioxidant capacity depends on the reversible dehydrogenation reaction [33]. Araloside A and L-ascorbic acid exerted synergistic antioxidant effects, probably due to the combination of the glycosylation activity of araloside A and the dehydrogenation reaction of ascorbic acid.

H_2_O_2_, a stable source of free radicals, is widely used in the construction of in vitro oxidative stress models. However, its effects depend on the cell type and may differ from those of other prooxidant agents. HepG2 cells were damaged by 10 mM H_2_O_2_, resulting in a cell viability of approximately 50%. H_2_O_2_ challenged the antioxidant defence system of HepG2 cells, causing ROS accumulation and protein and lipid damage in this oxidative stress model [34]. However, treatment of SH-SY5Y cells with 250 *μ*M H_2_O_2_ resulted in a cell death rate of approximately 50% [35]. Wang et al. used HTR8/SVneo cells treated with 200 *μ*M H_2_O_2_ for 12 h to investigate the protective effects of resveratrol against oxidative stress injury [36]. In contrast, the maximum oxidative damage was achieved when Chinese hamster cells were incubated with high-dose H_2_O_2_ for 5 min [37]. The optimal conditions for use in our study were 400 *μ*M H_2_O_2_ and 4 h (Figure 2), which agrees with the experimental results reported by Liu et al. [38]. The individual and combined protective effects of araloside A and L-ascorbic acid against H_2_O_2_-induced oxidative damage were evaluated by measuring cell viability and LDH release, and the SRs were screened. The results showed that araloside A, L-ascorbic acid, and their combination all increased the viability of H_2_O_2_-exposed HEK293 cells and decreased the release of LDH (Figure 3). These results reveal the potential synergistic effects of araloside A and L-ascorbic acid and demonstrate that araloside A in combination with L-ascorbic acid alleviates oxidative damage and maintains the integrity of the plasma membrane.

ROS and free radicals are by-products of normal cell metabolism and do not damage cells at low concentrations but contribute to interactions between cells [39]. However, at a too-high intracellular concentration, ROS and free radicals severely damage cells and tissues, which decreases the mitochondrial membrane potential and induces protein damage and DNA fragmentation [40]. In our experiments, we used the DCFH-DA fluorescent probe assay to evaluate the production and elimination of ROS in HEK293 cells. DCFH-DA is a nonfluorescent substance that can pass through the cell membrane; after entering a cell, this probe is delipidized into DCFH and further oxidized by ROS in the cell to form the highly fluorescent compound DCF [38]. The fluorescence intensity and density of DCF obtained by our experimental measurements reflected the effects of araloside A, L-ascorbic acid, and their combination on the production of intracellular ROS. The results showed that combined pretreatment with araloside A and L-ascorbic acid was more effective in inhibiting H_2_O_2_-induced intracellular ROS production than individual pretreatment (Figure 4). The protein carbonyl and 8-OHdG levels were used to represent the damaging effect of H_2_O_2_ on protein and DNA, respectively (Figure 6). Interestingly, compared to individual pretreatment, combined pretreatment more significantly reduced the protein carbonyl and 8-OHdG content. This result suggests that the combination of araloside A and L-ascorbic acid could protect HEK293 cells from H_2_O_2_-induced oxidative stress-related damage and that this effect might be partially due to the accelerated scavenging of intracellular ROS and reductions in protein and DNA damage.

Antioxidant defence systems include enzymes, including SOD, GSH-Px, and CAT, and nonenzymatic defences, including GSH, alpha tocopherol, and other antioxidants [41]. Antioxidant enzyme activity (SOD, GSH-Px, and CAT), T-AOC, and the GSH/GSSG ratio in HEK293 cells treated with H_2_O_2_ were decreased compared with those of the untreated cells. However, pretreatment with araloside A, L-ascorbic acid, and their combination significantly increased SOD, GSH-Px, and CAT activities, T-AOC, and the GSH/GSSG ratio (Figure 5). These results demonstrate that araloside A and/or L-ascorbic acid can be used as an exogenous antioxidant to increase the antioxidant defence capacity of cells and that their combination exerts significant synergistic effects. Pal et al. also reported that L-ascorbic acid can increase GSH-Px, SOD, and CAT activity and T-AOC [42], which is consistent with our results. MDA and LPO are important products of lipid peroxidation often used as biomarkers of oxidative stress levels. L-Ascorbic acid exerts protective effects against cellular oxidative stress-related damage by reducing the cellular MDA content [43]. The data from our study showed that araloside A effectively inhibited the increases in MDA and LPO contents and that combined pretreatment with araloside A and L-ascorbic acid was more effective in inhibiting these increases in the MDA and LPO contents than individual pretreatment with either compound (Figure 6). These results provided evidence demonstrating that araloside A and L-ascorbic acid synergistically inhibit cell lipid peroxidation.

The measurement of DPPH^·^, a stable source of nitrogen free radicals, is one of the most reliable and accurate methods to determine antioxidant capacity and is often used to study the antioxidant activity of active components in plants and animals [44]. Hydroxyl radicals are the most destructive ROS in foods and biological systems [45]. In our study, the capacity to scavenge DPPH^·^, ABTS^+^, hydroxyl, and superoxide anions was measured in vitro to evaluate the combined antioxidant potential of araloside A and L-ascorbic acid. The results also suggested that araloside A and L-ascorbic acid exhibit strong antioxidant capacity at lower concentrations.

Detection of the antioxidant capacity of active ingredients of plants requires determining antioxidant capacity both in vitro and in cells or animals, and whether a correlation between these systems exists must be studied. Ait Lahcen et al. evaluated the antioxidant capacity of a Moroccan *Cistus creticus* leaf extract by measuring DPPH^·^ and FRAP free radical scavenging abilities [46]. Santos et al. used assays to measure oxygen radical absorbance capacity, ferric reducing antioxidant power, and DPPH radical scavenging capacity to evaluate the antioxidant capacity of *Spathodea campanulata* [47]. Wang et al. evaluated the protective effects of araloside C against H_2_O_2_-induced oxidative stress by assessing the release of LDH, mitochondrial function, and bioenergetics in H9c2 cardiomyocytes [48]. The antioxidant activity of araloside A and L-ascorbic acid was studied in vitro, and their correlation in cellular pathways was analysed. The antioxidant status of HEK293 cells showed a significant and direct correlation with the ability of araloside A and L-ascorbic acid to scavenge DPPH^·^, ABTS^+^, hydroxyl, and superoxide anion radicals (Figure 8). The free radical scavenging ability was found to be positively correlated with SOD and CAT activity, the GSH/GSSG ratio, and T-AOC but negatively correlated with GSSG, MDA, LPO, LDH, protein carbonyl, 8-OHdG, H_2_O_2_, and ROS levels. Furthermore, the radical scavenging ability showed no correlation with cell viability or GSH-Px activity. In vitro antioxidant activity, particularly the DPPH^·^ free radical scavenging ability, might predict the effects of araloside A and L-ascorbic acid on the antioxidant status of cells.

In general, our manuscript still has some limitations. First, we evaluated only the synergistic antioxidant effects of araloside A and L-ascorbic acid, but determining whether these agents have synergistic anti-inflammatory and anticancer effects requires further discussion. Second, the indicators used to assess the protective effect of araloside A and L-ascorbic acid against protein and lipid damage are insufficient. The next step is to determine the signalling pathways that regulate their synergistic antioxidant mechanism, such as the Nrf2-Keap1 system.

## 5. Conclusions

The synergistic antioxidant activity of araloside A and L-ascorbic acid indicates that their combination has the potential to alleviate oxidative damage to food and biological systems. Studying the synergistic effects of natural antioxidants is important for the treatment of diseases related to oxidative stress. Our research results provide new ideas regarding the interaction between different plant active ingredients and the feasibility of the use of various dietary combinations involved in multiple pathways to regulate oxidative stress-induced damage and diseases.

## Figures and Tables

**Table 1:** Synergistic rate (SR) of the combination pretreatment of araloside A and L-ascorbic acid in H_2_O_2_-induced HEK-293 cells.

	Concentrations (araloside A+L-ascorbic acid)								
	EV			TV			SR		
	1	2	3	1	2	3	1	2	3
Cell viability (%)	78.37 ± 0.96	91.29 ± 1.94	95.44 ± 1.55	67.13 ± 1.17	77.08 ± 1.71	83.32 ± 1.98	1.07 ± 0.03^Cde^	1.18 ± 0.06^Ab^	1.14 ± 0.03^Be^
LDH (U/g pro)	405.29 ± 21.93	329.61 ± 11.23	230.39 ± 12.32	484.01 ± 11.35	349.36 ± 14.19	328.97 ± 16.07	0.84 ± 0.01^Bghi^	0.94 ± 0.02^Ae^	0.70 ± 0.01^Ck^
ROS	2.34 ± 0.06	1.79 ± 0.07	1.50 ± 0.02	2.79 ± 0.19	2.42 ± 0.07	1.91 ± 0.03	0.84 ± 0.02^Ahi^	0.73 ± 0.05^Cj^	0.78 ± 0.07^Bi^
CAT (U/mg pro)	10.18 ± 1.06	14.78 ± 0.64	17.50 ± 0.34	8.22 ± 0.26	9.98 ± 0.92	12.03 ± 0.68	1.24 ± 0.06^Ca^	1.48 ± 0.05^Aa^	1.45 ± 0.03^Bb^
SOD (U/mg pro)	16.02 ± 0.39	17.04 ± 0.81	18.54 ± 0.59	14.33 ± 0.71	14.39 ± 0.43	15.66 ± 0.61	1.12 ± 0.02^Cc^	1.18 ± 0.03^Bb^	1.88 ± 0.04^Aa^
GSH-Px (U/mg pro)	54.19 ± 1.82	65.01 ± 0.28	74.51 ± 0.58	50.74 ± 1.56	60.88 ± 0.98	70.22 ± 0.57	1.07 ± 0.03^Ae^	1.06 ± 0.04^Bd^	1.06 ± 0.05^Bf^
T-AOC (mM TE/g FM)	0.80 ± 0.36	0.82 ± 0.01	0.97 ± 0.03	0.67 ± 0.04	0.73 ± 0.02	0.83 ± 0.02	1.19 ± 0.02^Ab^	1.12 ± 0.01^Cc^	1.17 ± 0.03^Bd^
GSSG (*μ*mol/L)	32.79 ± 0.94	27.19 ± 1.04	20.12 ± 1.22	36.90 ± 1.67	33.11 ± 2.14	24.66 ± 1.14	0.88 ± 0.04^Ag^	0.82 ± 0.04^Bg^	0.81 ± 0.07^Ch^
GSH/GSSG	0.57 ± 0.01	0.78 ± 0.03	0.87 ± 0.04	0.52 ± 0.01	0.66 ± 0.02	0.71 ± 0.02	1.10 ± 0.02^Ce^	1.18 ± 0.02^Bb^	1.22 ± 0.03^Ac^
MDA (nmol/mg pro)	9.11 ± 0.23	8.55 ± 0.16	5.71 ± 0.15	10.69 ± 0.27	9.00 ± 0.06	7.06 ± 0.16	0.85 ± 0.03^Bgh^	0.95 ± 0.04^Ae^	0.80 ± 0.02^Ch^
H_2_O_2_ (mmol/g pro)	50.29 ± 0.34	37.20 ± 1.43	23.39 ± 0.31	54.16 ± 0.49	48.32 ± 1.05	30.30 ± 1.39	0.92 ± 0.06^Af^	0.76 ± 0.06^Ci^	0.77 ± 0.04^Bij^
LPO (*μ*mol/g pro)	16.13 ± 0.17	14.55 ± 0.13	10.71 ± 0.17	18.70 ± 0.35	17.03 ± 0.16	14.06 ± 0.25	0.86 ± 0.03^Agh^	0.85 ± 0.01^Bf^	0.76 ± 0.06^Cj^
Protein carbonyl (nmol/mg pro)	363.93 ± 5.39	213.39 ± 3.35	170.23 ± 6.88	383.30 ± 4.13	275.86 ± 5.81	197.28 ± 2.74	0.95 ± 0.01^Af^	0.77 ± 0.04^Bi^	0.86 ± 0.02^Cg^
8-OHdG	3.01 ± 0.03	2.83 ± 0.04	2.54 ± 0.02	3.73 ± 0.16	3.52 ± 0.05	2.94 ± 0.06	0.80 ± 0.03^Bi^	0.80 ± 0.01^Bh^	0.86 ± 0.02^Ag^

1: 0.5 *μ*M araloside A + 0.5 *μ*M L − ascorbic acid; 2: 5 *μ*M araloside A + 5 *μ*M L − ascorbic acid; 3: 10 *μ*M araloside A + 10 *μ*M L − ascorbic acid. The synergistic rate (SR) was calculated by the equation: SR = EV/TV. EV was the experimental value of the combination group. TV was the theoretical value of the combination group: TV = *a*∗*R*_*A*_ + *b*∗*R*_*B*_. Among them, *a* and *b* were the combined ratios of substance *A* and substance *B*, and *R*_*A*_ and *R*_*B*_ were the experimental results obtained when substance *A* and substance *B* are used separately. SR > 1 indicated the synergistic effects; SR < 1 indicated the antagonistic effects; SR = 1 indicated the addictive effects. In this table, SRs based on LDH release, ROS generation, GSSG content, MDA content, H_2_O_2_ level, LPO content, protein carbonyl level, and 8-OHdG level were less than 1, which represented the synergistic effects. LDH: the release of lactate dehydrogenase; ROS: the production of reactive oxygen species; CAT: the activity of catalase; SOD: the activity of superoxide dismutase; GSH-Px: the activity of glutathione peroxidase; T-AOC: the total antioxidant capacity; GSSG: oxidized glutathione; GSH/GSSG: the glutathione/oxidized glutathione ratio; MDA: the malondialdehyde levels; H_2_O_2_: the H_2_O_2_ levels; LPO: the lipid peroxidation contents; 8-OHdG: the 8-hydroxy-2-deoxy guanosine levels. Different uppercase letters indicate significant differences between SR values in the same row (*p* < 0.05); different lowercase letters indicate significant differences between SR values in the same column (*p* < 0.05).

## Data Availability

Data will be available on request.
